# Supplementary material for: Would you respect a norm if it sounds foreign? Foreign-accented speech affects decision-making processes
Source: PLoS One. 2022 Oct 5;17(10):e0274727. doi: 10.1371/journal.pone.0274727 (PMC9534425; doi:10.1371/journal.pone.0274727)
Supplement: S1 Table — (DOCX) [file pone.0274727.s001.docx]

**Supplementary Materials**

S1 Table: *Estimates, standard error, t-values, and p-values of the predictor Accent (native, foreign) on the four emotions (anger, disgust, sadness, and fear)*

|  | Estimate | Std. Error | t-value | p-value |
| --- | --- | --- | --- | --- |
| Anger | | | | |
| Intercept | 20.05 | 3.21 | 6.22 | .001 |
| Accent | 6.62 | 4.58 | 1.44 | 0.15 |
| Disgust | | | | |
| Intercept | 9.04 | 2.19 | 4.11 | .001 |
| Accent | 3.60 | 3.13 | 1.15 | .25 |
| Sadness | | | | |
| Intercept | 42.49 | 3.61 | 11.76 | .01 |
| Accent | -2.20 | 5.15 | -.42 | .66 |
| Fear | | | | |
| Intercept | 18.49 | 3.12 | 5.91 | .01 |
| Accent | 1.80 | 4.45 | .40 | 0.68 |
