# Supplementary material for: Would you respect a norm if it sounds foreign? Foreign-accented speech affects decision-making processes
Source: PLoS One. 2022 Oct 5;17(10):e0274727. doi: 10.1371/journal.pone.0274727 (PMC9534425; doi:10.1371/journal.pone.0274727)
Supplement: S2 Table — Means (SDs) refer to the rating attributed to the norms in the pre-test. The structure of the sentences was adapted to match the Spanish translation of the questions ‘How likely are you to respect the norm…’ (Respect variable) and ‘How wrong is it to…’ (Wrong social variable). (DOCX) [file pone.0274727.s002.docx]

S2 Table: *English translation of the* *everyday social norms included in Task 1 of Experiment 2 (norms marked with ‘*’ were not included because of a low rating in the pre-test). Means (SDs) refer to the rating attributed to the norms in the pre-test. The structure of the sentences was adapted to match the Spanish translation of the questions ‘How likely are you to respect the norm…’ (Respect variable) and ‘How wrong is it to…’ (Wrong social variable)*

| Social norms | Mean (SD) |
| --- | --- |
| Taking credit for others’ work. | 9.79 (0.57) |
| Littering on the street or in public places. | 9.71 (0.61) |
| Not offering to help after an accident. | 9.43 (1.34) |
| Taking the last seat on a crowded bus. | 9.07 (1.63) |
| Selling someone a defective car | 9.00 (1.92) |
| Driving after drinking alcohol. | 8.93 (1.94) |
| Not paying for the ticket on public transportation. | 8.29 (2.58) |
| Not helping someone pick up their dropped papers. | 7.50 (2.17) |
| Cutting in line when in a hurry. | 7.29 (1.81) |
| Be mean to someone you don’t like. | 7.29 (1.81) |
| Keeping excess-change at a store. | 7.14 (2.87) |
| Parking in a handicap spot. | 7.07 (2.23) |
| Ignoring a homeless that needs food* | 7.00 (1.79) |
| Lying to get a special offer* | 6.14 (2.44) |
| Not helping somebody in need* | 5.45 (2.19) |
