# Supplementary material for: Would you respect a norm if it sounds foreign? Foreign-accented speech affects decision-making processes
Source: PLoS One. 2022 Oct 5;17(10):e0274727. doi: 10.1371/journal.pone.0274727 (PMC9534425; doi:10.1371/journal.pone.0274727)
Supplement: S3 Table — (DOCX) [file pone.0274727.s003.docx]

S3 Table: *Estimates, standard error, t-values, and p-values of the predictor Accent (native, foreign-Indo, foreign-Arab) in interaction with Accent Strength, Comprehensibility and Education on Respect.*

|  | Estimate | Std. Error | t-value | p-value |
| --- | --- | --- | --- | --- |
| Accent Strength | | | | |
| Intercept | 86.95 | 2.49 | 34.90 | <.001 |
| Accent_NativeForeign-Indo_ | 3.17 | 2.33 | 1.35 | 0.17 |
| Accent_NativeForeign-Arab_ | -0.92 | 2.35 | -0.39 | .69 |
| Accent Strength | <-0.001 | .04 | -0.01 | .99 |
| Accent strength by Accent_NativeForeign-Indo_ | -0.10 | 7.23 | -1.53 | .12 |
| Accent strength by Accent_NativeForeign-Arab_ | -0.03 | .06 | -0.55 | .58 |
| Comprehensibility | | | | |
| Intercept | 88.32 | 2.89 | 30.46 | <.001 |
| Accent_NativeForeign-Indo_ | .19 | 2.61 | .07 | .94 |
| Accent_NativeForeign-Arab_ | -1.45 | 2.76 | -0.52 | .59 |
| Comprehensibility | .07 | .10 | .70 | .48 |
| Comprehensibility by Accent_NativeForeign-Indo_ | -0.17 | .12 | -1.41 | .15 |
| Comprehensibility by Accent_NativeForeign-Arab_ | -0.15 | .11 | -1.31 | .19 |
| Education |  |  |  |  |
| Intercept | 87.01 | 2.22 | 38.75 | <.001 |
| Accent_NativeForeign-Indo_ | 1.47 | 1.91 | .77 | .44 |
| Accent_NativeForeign-Arab_ | -0.39 | 1.99 | -0.20 | .84 |
| Education | -0.006 | .08 | -0.08 | .93 |
| Education by Accent_NativeForeign-Indo_ | .02 | .10 | .24 | .81 |
| Education by Accent_NativeForeign-Arab_ | .12 | .10 | 1.27 | .20 |
