# Supplementary material for: Would you respect a norm if it sounds foreign? Foreign-accented speech affects decision-making processes
Source: PLoS One. 2022 Oct 5;17(10):e0274727. doi: 10.1371/journal.pone.0274727 (PMC9534425; doi:10.1371/journal.pone.0274727)
Supplement: Attachment — Submitted filename: Response to Reviewers.docx [file pone.0274727.s004.docx]

Dear Editor,

Thank you very much for considering our manuscript entitled “*Would you respect a norm if it sounds foreign? Foreign-accented speech affects decision-making processes*” for publication in the Journal *Plos ONE*. We would like to thank the reviewer for their positive, supportive, and constructive feedback.

Following their comments, we have carefully revised our manuscript. In particular, we have changed three substantial parts of our manuscript. First, we have justified why we classified the social norms related to Covid as less emotional and with a less intrinsic cultural relation than everyday norms. Second, we have better justified the inclusion of the Arabic accent by stressing the fact that not only is it perceived as negatively but it is also the most common type of foreign accent in Spain. Third, we have included additional theoretical background to better explain the difference between social categorization and stereotypes. The main changes are detailed below and highlighted in the text, whereas minor changes have been directly included in the text.

Finally, we would like to thank you and the Reviewer once again for your useful comments, we believe the changes have resulted in a much stronger article. We hope that you will share this opinion.

Yours sincerely,

Luca Bazzi on behalf of the authors.

**Response to the Reviewer’s comments**

1. **There are two main points in the manuscript that seem to me to be poorly justified:
   On the one hand, the second study is justified in part because more established social norms are supposedly acquired in interactions with native speakers and are more emotional. But I don't see why social norms related to COVID would not be acquired in interactions with native speakers or would be less emotional.**

*We thank the reviewer for this comment, we agree that our justification may have been misleading, especially the sentence ‘Importantly, these norms are usually learned in a native language context and therefore, associated to it (65). We have deleted this sentence.*

*The reviewer is right that both established social norms and norms related to COVID are learnt in interaction with native speakers. However, as we now explain on page 17, ‘in contrast to the norms rapidly imposed in response to the COVID pandemic, moral and social norms are learned gradually from childhood, and have an intrinsic cultural and linguistic link. Therefore, and most importantly, social norms are associated to the cultural and linguistic context of learning.’*

*Moreover, we now better justify the rationale behind Experiment 2 by explaining that social and moral norms are linked to autobiographical memories, which are considered language specific, and hence, if the language of retrieval matches the language of encoding, retrieval is facilitated. Given that accent is an aspect of language, we draw a parallel between foreign language and foreign accent and motivate our hypothesis. It reads as follows:*

As mentioned in the Introduction, Geipel and colleagues (2015) have shown that social norms processed in a foreign language provoked less harsh judgements than those processed in a native language. The authors attempted to justify their results by arguing that the wrongness attitude towards a social or moral transgression may be linked to autobiographical memories and social-cultural learning process. Importantly, memories are considered language specific, and hence, if the language of retrieval matches the language of encoding, retrieval is facilitated. In other words, they suggested that since social norms are learned early in life by relatives or caregivers, they are usually encoded in one’s native language, hence, recalling these norms in a native language is easier than doing so in a foreign language. The reduction of sensibility to norms is also visible through the use of stronger swearwords in a foreign language, which suggests that social norms are less accessible and less salient than in a native language. Given that accent is an aspect of language, it is possible that norms encoded in a native accent would be easier to recall if processed in the same native accent than in a foreign accent (see (32) for a similar proposal). To test this hypothesis, we adapted Geipel and colleagues’ (2015) study. (p. 18)

2. **On the other hand, the other justification for the second study is based on the idea that participants would be more familiar with the Arabic accent, and this might lead to more negative biases. However, neither of these two issues ends up being properly demonstrated (less than half of the participants correctly recognized the accent of the speaker; nor did they have more negative biases when listening to this speaker).**

*We thank the review for the comment, which is two-fold. First, regarding the familiarity of the accents, it is indeed true that less than half (49%) of the participants identified the foreign accent as Arabic, but, only 6% of the participants correctly identified the Indonesian speaker as Asian. To support our impression that the Moroccan accent is more familiar than the Indonesian in Spain, we added statistics from the Spanish National Institute of Statistics. It reads as follows:*

We consider the Moroccan accent as more familiar than the Indonesian accent because the Moroccan nationality is the first foreign nationality in Spain, according to the Spanish National Institute of Statistics, representing 16% of the foreign population in the country (in contrast, the first Asian nationality is Chinese and represent only 4%). (p. 9)

*Second, regarding the negative bias, in the first version of the manuscript, we already mentioned a recent study that has shown that the Arabic population is the least liked population in Spain (see p. 9). Thanks to the reviewer’s suggestion in point 14, we now also report additional results from our own study that reveal that our participants assessed the Moroccan speaker as significantly less educated than the Indonesian speaker (p. 22). Given that no information was provided about the speaker’s education, we attribute this score to the negative perception towards Arabic people.*

3. **At some points, authors do not follow the journal’s referencing style. Please review it.**

*We thank the reviewer for pointing out the mistake. We have updated all the references to the journal’s style.*

4. **Abstract, lines 30-31: “or in a foreign accent unfamiliar to our participants to avoid stereotypes”. How do you know that the unfamiliar accent did not activate stereotypes towards foreign-accented speakers (outgroup members)?**

*Please see response to comment 5.*

5. **As you write in the Introduction (lines 121-124): “The negative perception of a foreign-accented speaker is not only due to the disfluency of speech, but also to the social categorization of the speaker. Indeed, by the simple fact of saying ‘hello’, a speaker reveals her social background and is rapidly categorized as an in- or out-group member. This is an issue that may be addressed throughout the manuscript.**

*We agree with the reviewer that more detailed explanation about the mechanisms underlying the perception of foreign speakers was required. We have added this information at various points throughout the manuscript (see p. 6, 9, 33). Particularly, the main explanation that addresses the issue raised by the reviewer in comments 4 and 5 can be found on page 6. It reads as follows:*

The negative perception of a foreign-accented speaker is not only due to the disfluency of speech, but also, and primarily, to the social categorization of the speaker. According to Mai and Hoffman (12), social categorization is a process that takes place even before the speaker’s message is decoded. Indeed, by the simple fact of saying ‘hello’, a speaker reveals her social background and is rapidly categorized as an in- or out-group member (40). This process is implicit and independent of stereotypes, as it is observed even in young children who prefer to befriend with native speakers than foreign speakers (59). This categorization may, subsequently, trigger (positive or negative) stereotypes associated with the speaker’s culture (51,52). Hence, the impression of a foreign-accented speaker is first based on the implicit process of social categorization, after only a few seconds of communication. Then, this initial impression may be positively or negatively nuanced by stereotypes and language processing, processes that are therefore secondary and more complex than social categorization since they develop in the time course of communication. Importantly, the simple fact of being considered a member of a different group generates some psychological distance that may affect how the information coming from the speaker is interpreted (53,54).

6. **Sub-section “Foreign-accented speech and emotion processing”, lines 88-95. I would recommend the authors to check a recently published paper in which this perspective on the effects of foreign-accented speech on lexical-semantic access is discussed: Romero-Rivas, C., & Costa, A. (2022). On the flexibility of the sound-to-meaning mapping when listening to native and foreign-accented speech. Cortex, 149, 1-15. According to this paper, and following more recent models of speech processing (e.g., Hickok & Poeppel, 2007; Gwilliams, Poeppel, et al., 2018), non-standard speech signals (such as the ones produced by foreign-accented speakers) do not necessarily impair access to lexical-semantic information. Therefore, the effects obtained by Hatzidaki et al. (2015) (and by other authors in other studies) may be alternatively explained by how the acoustic properties of the native vs. foreign-accented words (e.g., longer duration of the words in the latter vs. former) might artificially affect the ERPs. I’d just like to suggest that one should consider the possibility that the effects found by these authors could be explained by the properties of the acoustic signal (such as the duration of the stimuli), and not so much by the identity of the speakers.This comment does not only apply to Hatzidaki et al., but also to the papers that are reviewed below in the next sections.**

*We agree with the reviewer that it is an alternative explanation, and we now mention it in our manuscript.*

Alternatively, as suggested by an anonymous reviewer, Hatzidaki et al.’s study, and in general, in the foreign-accent literature, the difference between the native and foreign accent conditions may be due to the acoustic properties of the speakers. Discussing this alternative goes beyond the scope of the study but the interesting reader can refer to recent articles that addressed the question. (p. 5)

7. **Participants sub-section, lines 155-157: “Given that familiarity with other languages may affect accent’s perception (5,55,56), participants’ knowledge of other languages was also controlled for to avoid differences across conditions”.
   Could you please clarify what you meant here, and how did you control for this variability across participants?**

*Following the reviewer’s comment, we have added more information to better explain how we controlled for the participants familiarity of foreign languages. We were particularly interested in their knowledge of the foreign speakers’ native language, i.e., Arab and Indonesian since it has been shown to affect language processing. None of them had knowledge of these languages. It now reads as follows:*

Given that familiarity with other languages may affect accent’s perception and language processing (5,60,61), participants’ knowledge of other languages, especially Arab and Indonesian, was also controlled for using a linguistic background questionnaire. All participants had knowledge of English from school. In addition, 7.3% could communicate in French, 2.5% in Italian and 1.1% in German. None of them spoke Arab or Indonesian. (p. 8)

8. **Speakers sub-section, lines 174-175: “In Experiment 1, we sought to avoid an effect of stereotype and therefore included an unfamiliar accent, Indonesian”. It is not clear to me whether including an, a priori, unfamiliar accent, would prevent participants for activating stereotypes. For instance, an unfamiliar accent may activate outgroup membership associations, and potentially generate negative biases towards that person.**

*Please, see response to comment 5. Additionally, we would like to mention that we agree with the reviewer that the effects of the identity of the speaker, especially the differences between social categorizations and stereotypes are an important question. We are currently undertaking experiments in our laboratory to better understand this phenomenon and disentangle the effects these two processes may have on foreign-accented speech comprehension and our decisions.*

9. **Speakers sub-section, lines 175-176: “In Experiment 2, we included a familiar accent, usually perceived negatively in Spain, Moroccan”. The problem is that, later in the same paragraph, you mention that only 49% of participants were able to correctly recognize the speaker’s accent. Thus, any conclusion about the activation of stereotypes specifically associated with this accent is difficult to sustain.**

*Please, see response to comment 6.*

10. **Lines 250-252: Isn’t it redundant to say twice that the Native accent condition was dummy-coded as 0?**

*We thank the reviewer for catching this error. We have removed the repetition on p.12.*

11. **Lines 297-300: Why didn’t you carry out the same analysis on Efficiency?**

*We did carry out this analysis, but we forgot to report it in the first version of the manuscript. We thank the reviewer for pointing out this mistake. We now describe the data analysis on p. 13 and the results can be found on p. 16.*

12. **Line 389: It is not clear what you meant by “Task 1”. Did participants complete more than one task?**

*The participants did not complete more than one tasks so we have removed the misleading information.*

13. **Lines 419-424: You should also report whether there were any differences between the foreign-Indo and foreign-Arab accents in terms of Accent Strength and Comprehensibility.**

*We answer questions 13 and 14 together.*

14. **Same applies to Education (lines 427-429).**

*We appreciate the reviewer’s comment, and we would like to mention that we did not include post-hoc tests (native vs. foreign-Indo + native vs. foreign-Arab + foreign-Indo vs. foreign-Arab) in our ANOVA analyses nor in the Linear regressions, but we used planned contrasts instead (native vs. foreign-Indo + native vs. foreign-Arab). The reason behind this decision is based on the fact that our main research question focuses on the impact of foreign accent on native speakers’ behavior, and not on the differences between foreign accents/speakers. Furthermore, this contrast was not part of our original hypotheses when designing the experiment, thus, we believe that presenting the results between the two foreign accent conditions (foreign-Indo vs. foreign-Arab) would go beyond the scope of our study. However, we agree that this is a very interesting topic that we are currently investigating in another specific study.*

*Nevertheless, we took into consideration the reviewer’s comments and included post-hoc tests with all possible contrasts for the linguistic and social variables (see p. 22). We agree that reporting these results provide the reader with a better picture of the perception of the speakers and may help better understand the results we obtained in the main analyses (likelihood to respect a norm, wrongness, and efficiency).*

15. **Lines 435-441: Before reporting the individual comparisons between specific conditions, you should carry out/report the analysis for the main factor Accent (both when analyzing Respect and Wrong). This can be done by dummy coding the three levels of the main factor (e.g., Native Accent = 0; Foreign-Indo = 1; Foreign-Arab = 2; since you were expecting larger effects in the latter condition). Same applies to the analyses on Emotion. These analyses could be either linear mixed models or ANOVAs.**

*Please, see response to comments 13 and 14.*

16. **Lines 470-478: I am sorry, but I am not able to understand this analysis. I believe that you entered each potential comparison between the native and the foreign accents as predictors in the model; then, emotion and the interactions between emotion and the comparisons were also entered into the model. Why did you do this? Is there any particular reason to justify this decision? Possibly, a more appropriate way to conduct this analysis would be to include the main effect of accent, the main effect of emotion, and the interaction between the two factors.**

*We did enter Accent as a main fixed effect in the model, but we used planned contrasts so that we had two comparisons or contrasts. However, we understand the review’s confusion as it was not very clearly described in the first version of the manuscript. For that reason, we have modified Tables 8 and 9 to reflect the two contrasts included in the analysis. In particular, we included the following labels to indicate the two contrasts: Accent_NATIVEVSFOREIGN-INDO_ and Accent_NATIVEVSFOREIGN-ARAB_. The first contrast compared participants’ decisions on the native accent versus the foreign-Indo accent, while the second contrast compared decisions on the native accent versus the foreign-Arab accent. We now added this information explicitly to the manuscript on p. 21.*

17. **Then, if you find a main effect of accent, you may proceed and check the post-hoc comparisons. Same would happen with the interaction between factors. Also, at no point you explore the potential difference between the two foreign accents, and that may be informative as well.**

*As mentioned in the response to point 14, we did not explore the differences between foreign accents because this comparison goes beyond the main purpose of our investigation. Our research questions focus on the effect of a foreign accent on native speakers’ behavior, but not on the differences across foreign accents, which would respond to another research question. We agree with the reviewer that the impact of the speaker’s nationality and identity on our decisions is an extremely interesting topic that we are currently investigating in a different study.*

18. **Same applies to the analysis reported in lines 491-498. These analyses could be either linear mixed models or ANOVAs.**

*Unfortunately, it was not possible to conduct linear mixed effects models as the participants were not exposed to all the norms in the experimental design.*

19. **Also, if the interaction between Accent and Emotion ends up being significant, I think that readers would appreciate a graphic representation of this interactions, so they can properly interpret/follow the results.**

*We have added the graphic representation of the interaction effect between Accent and Emotion on Respect and Wrong on p. 26 and 28.*

20. **Lines 513-517: These conclusions (and the ones in the next section) may change after carrying out the analyses I suggest above.**

*Please, see response to comments 13 and 14.*

21. **Lines 574-578: If I am not wrong, you did not include participants’ fluency ratings in your regression models. Therefore, I do not understand why you say that “this variable did not influence participants’ decisions”.**

*We did conduct this analysis; the reviewer can find it on page 27 and the results in Table S3 in the Supplementary Materials. No effect of Comprehensibility on Respect was found. Hence, we included the significant linguistic and social variables, i.e., Accent Strength, Comprehensibility, and Education in the model in interaction with Accent. None of these variables were significant (see Table S3 in the Supplementary Materials).*

22. **Lines 588-598: These conclusions are interesting, but my feeling is that the results went in the opposite direction of your hypotheses, didn’t they?** **If the Indonesian speaker was perceived as more pleasant, one may expect to observe positive effects on decision-making; and the opposite may be expected from the Moroccan speaker, who was perceived as less educated. However, the pattern of results is contradictory with the expectations. Have you thought about any explanation for this pattern?**

*We thank the reviewer for this interesting comment, and we agree that the perception of each speaker is not directly reflected in the main results (e.g., Respect). However, it is reflected in the emotion related to the speaker, and we believe the way it indirectly modulated participants’ decisions converges with our hypothesis. This is explained on page 31-32.*

Interestingly, the emotions felt by the participants varied according to the speakers. The unfamiliar accent (Indonesian) provoked a more intense emotional response than the native accent, whereas the negative accent (Moroccan) provoked a less intense one. This pattern was reflected in the way emotion modulated participants’ decisions. Indeed, although we found that the more intense the emotional response, the higher the level of wrongness for the three accents, this effect was significantly larger for the Indonesian accent. In contrast, while the level of respect was similarly modulated by emotion in the native accent and Indonesian accents, it was not so in the Moroccan accent. Hence, accent seems to be a linguistic aspect that modulates emotionality; however, this modulation may be driven by the language attitudes related to the speaker, which leads us to our next points.
