# Supplementary material for: Would you respect a norm if it sounds foreign? Foreign-accented speech affects decision-making processes
Source: PLoS One. 2022 Oct 5;17(10):e0274727. doi: 10.1371/journal.pone.0274727 (PMC9534425; doi:10.1371/journal.pone.0274727)
Supplement: Attachment — Submitted filename: ResponseReviewers_R2.docx [file pone.0274727.s005.docx]

Dear Editor,

We would like to thank you and the Reviewer for your comments according to which we have revised the manuscript entitled “*Would you respect a norm if it sounds foreign? Foreign-accented speech affects decision-making processes*”.

Changes have been marked in the main text. Our detailed responses to major changes are provided below.

We believe that, thanks to the Reviewer’s comments, the manuscript has greatly improved and is now suitable for publication in *Plos ONE*.

Yours sincerely,

Luca Bazzi on behalf of the authors.

**********

Reviewer #1: I would like to congratulate the authors for the revision of their manuscript. I believe that they present a more robust version this time, but I still found some relevant issues that should be addressed before the article can be published. Please find more detailed comments below:

*We thank the reviewer for the positive comments and we hope the revised version addresses all her/his remaining concerns.*

- I still detected some inconsistencies in the referencing style (e.g., lines 80, 120, 145-146). Please recheck the document to make sure you are following the journal's referencing style.

*We apologies for missing inconsistencies that were generated by the automatic reference manager programme, we have now manually revised all the references to follow to the journal’s referencing style.*

- Line 165, typo: it should read “Experiments” rather than “Experiment”.

*Corrected*

- Lines 344-347: Based on this comment of the authors (i.e., that stereotypes associated with a group might affect the decision to accept a norm; which is repeated in the Discussion, lines 578-579), I still consider that it would be important to compare the two foreign accents: if indeed the accent effect found by the authors is due to the stereotype generated by a recognizable group (i.e., Moroccan), the expected result would be that the Moroccan accent produces a greater modulation of respect for the norm than the Indonesian accent. This could be addressed by using the Accent factor (with three levels: native, foreign-Moroccan, foreign-Indonesian) as a fixed factor in the analysis, or by adding the foreign-Moroccan vs. foreign-Indonesian accent comparison to the model. You have the data available, so it would be a shame not to perform this analysis.*.*

*We followed the reviewer's suggestion and compared the foreign-Indonesian with the foreign-Moroccan accent on the dependent variables Respect and Wrong. We reformulated this in the data analysis section (see p. 20-21):*

*We included this explanation in the manuscript:*

*“Accent was first treatment-coded with the native accent at the reference level (coded as 0). This created two different contrasts. The first contrast compared participants’ decisions on the native accent versus the foreign-Indo accent, while the second contrast compared decisions on the native accent versus the foreign-Arab accent. In addition, Accent was treatment-coded with the foreign-Indo accent at the reference level (coded as 0) such that it was also possible to directly compare the participants’ decisions on the foreign-Indo accent versus the foreign-Arab accent.”*

*The results showed no difference between the two foreign accents for Respect (β*_Foreign-IndovsForeign-Arab_= -3.17, *SE*= 1.85, *t*= -1.71, *p*= .08) *nor for Wrong (β_Foreign-IndovsForeign-Arab_= -3.34, SE= 2.32, t= -1.44, p= .15).*

*We have included this information on p. 23 in the results section and have adjusted the discussions. We particularly emphasize that, although no differences across accent conditions were directly observed on Respect and Wrongness, differences were observed on Emotion. Importantly, our design does not allow us to disentangle whether these differences were triggered by social categorization, stereotypes, the difficulty in comprehend the speech or acoustic differences, as mentioned in the discussion of Experiment 2 and further considered in the Discussion. Further studies will need to address this question.*

- Line 369: “(…) they suggested that since social norms are learned early in life by relatives or caregivers…”. It should read: “(…) they suggested that since social norms are TAUGHT early in life by relatives or caregivers…”.

*Corrected*

- Line 468: “foreign-Arab was significantly less educated than the foreign-Indo speaker”. It should read: “foreign-Arab was RATED AS significantly less educated than the foreign-Indo speaker”.

*Corrected*

- Figure 1: My apologies, but I do not understand this figure. In the text you state: “a multiple linear regression demonstrated a significant interaction between Accent and Emotion on Respect for the second contrast (βNativevsForeign-Arab= -0.06, SE= .01, t= -3.78, p< .001; Fig 1), but not for the first contrast (p> .1). This indicates that the level of respect increased when the level of emotion increased for the native accent, while the pattern remained stable for the foreign-Arab accent”. However, the pattern of the relationship between Accent and Emotion is the same (or extremely similar) in the three regression curves you present in Figure 1. Perhaps if, in addition to the regression slope, you showed the individual points for each participant, the picture would be easier to read. Additionally, presenting the two regression slopes of the same comparison in the same graph would also make it easier to interpret this effect (that is, at the end, you would have two graphs in Figure 1: one showing the regression slopes for the native and foreign-indo accents, and another one showing the regression slopes for the native and foreign-arab accetns). Same applies to Figure 2.

*Following the reviewer’s suggestion, we have modified Figure 1 and Figure 2. Both now display the direct comparisons between the three regression slopes for the native, foreign-Indo accents, and foreign-Arab accents, as well as the* *individual data points for each participant. We have chosen to add all three lines in one figure, as we now statistically compare all three accents with each other. These can be found on p. 27 and p. 29, respectively.*

- I do not understand why you find opposite results in different models: when analyzing Respect and Wrong across Accents (p. 23 and Table 6), you do not find accent effects. However, when you add Emotion to the model (and the interaction terms), you do find simple effects of Accent for Respect and Wrong (NativeVSForeignIndo and NativeVSForeignArab, respectively). These results (and, more concretely, the effect of Accent on Respect for the contrast NativeVsForeignIndo) would also contradict the results of Experiment 1. That makes me think that something failed in these latter models. Please review that the data and models are appropriate.

*In the models reported on p. 23, only Accent is added as predictor in the model. In these models, the three different contrasts can be directly interpreted (native vs. foreign-Indo; native vs. foreign-Arab; foreign-Indo vs. foreign-Arab), as there is only one category of the predictor on the intercept (either native or foreign-Indo, depending on the reference level). In this case, we did not find main effects of Accent.*

*However, when an additional predictor is added to the same model, that second predictor also influences the value of the intercept and the estimates of the other predictors. In this case, the predictor Emotion was added to the model. Importantly, we centered that predictor so in this case possible differences between accents are evaluated at the mean of emotion and are therefore simple effects. We now made this clearer on p. 26-*

- The names for the contrasts are different in Tables 9 and 10. Please revise.

*Corrected*

- Lines 563-567: this paragraph should be located at the end of the previous section, as it is summarizing the results of Experiments 2, not the results of the cross-experiment analyses. Furthermore, in the latter models you presented, you do find simple effects of Accent on Respect and Wrong. Please revise your analyses and the text, if needed.

*We have moved the paragraph and revised the text to make clearer that we did not observe a direct effect of accent on the likelihood to respect a norm or on the evaluation of the wrongness of its transgression, only a simple effect of Accent when emotion was held constant at the mean. (p. 30)*

- Cross-experimental analyses: I do not follow why you only use the native accent in these analyses. I believe that it would be more interesting to carry out 2-way ANOVAs with Experiment (1 vs. 2) and Accent (native vs. foreign-Indo) for each DV.

*The cross-experimental analyses served exclusively as a control check of our manipulation and hypothesis that individuals are more sensitive to everyday social norms learned from childhood than to new social norms imposed due to the Covid-19 pandemic. As mentioned in the introduction of Experiment 2, social norms have an intrinsic cultural and linguistic link and they are associated to the cultural context of learning, hence, we checked our manipulation for the native accent only because the results for the foreign accents could be biased by a different learning context. We have added this explanation (p. 30)*

- Lines 583-585: But you find that Accent has an effect on Respect/Wrong in Experiment 2 (simple effect), in the model in which you included Emotion and the interaction between Accent and Emotion. As I said before, please revise the data and models, and if the results are still the same, you may change some aspects of the Discussion.

*Please, see the response to the previous similar comments.*
